# Physiological Action of Medicines

**Published:** 1888-03

**Authors:** 


**Physiological Action of Medicines.** By LOUIS STARR, M. D., JAMES B. WALKER, M. D., and W. M. POWELL, M. D. Third edition. Enlarged. P. Blakiston, Son & Co.: Philadelphia.

This little book contains an outline of the action of some of the most important drugs, and is intended to be an epitome to assist the medical student in the study of pharmacology. The work would be of but little value, except in colleges, where the classification given by the authors is adopted.

